# Epidemiological characterization of symptomatic and asymptomatic COVID-19 cases and positivity in subsequent RT-PCR tests in the United Arab Emirates

**DOI:** 10.1371/journal.pone.0246903

**Published:** 2021-02-12

**Authors:** Rami H. Al-Rifai, Juan Acuna, Farida Ismail Al Hossany, Bashir Aden, Shamma Abdullah Al Memari, Shereena Khamis Al Mazrouei, Luai A. Ahmed

**Affiliations:** 1 Institute of Public Health, College of Medicine and Health Sciences, United Arab Emirates University, Al Ain, United Arab Emirates; 2 Department of Epidemiology and Public Health, College of Medicine and Health Sciences, Khalifa University, Abu Dhabi, United Arab Emirates; 3 Abu Dhabi Public Health Center, Abu Dhabi, United Arab Emirates; Faculty of Science, Ain Shams University (ASU), EGYPT

## Abstract

The coronavirus disease 2019 (COVID-19) cases could be symptomatic or asymptomatic. We (1) characterized and analyzed data collected from the first cohort of reverse transcriptase polymerase chain reaction (RT-PCR)-confirmed COVID-19 cases reported in the Emirate of Abu Dhabi, United Arab Emirates, according to the symptomatic state, and (2) identified factors associated with the symptomatic state. The association between the symptomatic state and testing positive in three subsequent RT-PCR testing rounds was also quantified. Between February 28 and April 8, 2020, 1,249 cases were reported. Sociodemographic characteristics, working status, travel history, and chronic comorbidities of 791 cases were analyzed according to the symptomatic state (symptomatic or asymptomatic). After the first confirmatory test, the results of three subsequent tests were analyzed. The mean age of the 791 cases was 35.6 ± 12.7 years (range: 1–81). Nearly 57.0% of cases were symptomatic. The two most frequent symptoms were fever (58.0%) and cough (41.0%). Symptomatic cases (mean age 36.3 ± 12.6 years) were significantly older than asymptomatic cases (mean age 34.5 ± 12.7 years). Compared with nonworking populations, working in public places (adjusted odds ratio (aOR), 1.76, 95% confidence interval (95% CI): 1.11–2.80), healthcare settings (aOR, 2.09, 95% CI: 1.01–4.31), or in the aviation and tourism sectors (aOR, 2.24, 95% CI: 1.14–4.40) was independently associated with the symptomatic state. Reporting at least one chronic comorbidity was also associated with symptomatic cases (aOR, 1.76, 95% CI: 1.03–3.01). Compared with asymptomatic cases, symptomatic cases had a prolonged duration of viral shedding and consistent odds of ≥2 positive COVID-19 tests result out of the three subsequent testing rounds. A substantial proportion of the diagnosed COVID-19 cases in the Emirate of Abu Dhabi were asymptomatic. Quarantining asymptomatic cases, implementing prevention measures, and raising awareness among populations working in high-risk settings are warranted.

## Introduction

Coronavirus disease 2019 (COVID-19) emerged in Wuhan, China, in December 2019 [1]. Due to sustained human-to-human transmission, COVID-19 has rapidly spread globally in >215 countries, affecting over 77 million people and causing over 1.7 million deaths, as of December 22, 2020 [2]. Approximately 80% of COVID-19 cases are asymptomatic or mild, 15% are severe and require oxygen, and 5% are critical infections that require ventilation [3]. Asymptomatic infection refers to the identification of viral nucleic acid by reverse transcriptase polymerase chain reaction (RT-PCR) in patients not displaying typical clinical symptoms.

One of the characteristics responsible for the difficulty in controlling the COVID-19 pandemic is the infectious status of those who are asymptomatic or very mildly symptomatic [3]. Asymptomatic COVID-19 infections reported having a similar viral load as those of symptomatic infections [4,5]. Contracting viral infection without showing clinical symptoms is highly likely to occur in the event of close contact with confirmed cases. In Boston, 88% of COVID-19 positive cases were asymptomatic [6]. In Japan, 30.8% of Japanese citizens evacuated from Wuhan [7] and 51.7% of COVID-19 cases from the “Diamond Princess” cruise [8] were asymptomatic. The viral RNA can be detected in the respiratory secretions of asymptomatic patients for no less than 3–5 days [4]. Thus, a mild or asymptomatic COVID-19 case can potentially transmit the virus to other people without any awareness.

The United Arab Emirates (UAE) is described as a melting pot of cultures with a total population of 10 million, 85% of whom are expatriates. The UAE is burdened with a high prevalence of noncommunicable diseases, mainly obesity and diabetes mellitus (DM) [9,10]. The prevalence of overweight and obesity among expatriates in the UAE is 43.0% and 32.3%, respectively [10]. Furthermore, the prevalence of DM and hypertension is 11.8% and 49.2% among nonobese individuals and 23.5% and 43.2% among obese expatriates residing in the UAE, respectively [10]. This high burden of noncommunicable diseases may contribute to increased susceptibility to COVID-19.

The first COVID-19 case in the UAE was detected on January 29, 2020. By December 22, 2020, 195,878 COVID-19 cases and 642 deaths due to COVID-19 had been reported in the UAE [2]. In addition to contact tracing and testing, public health measures including mass testing of random samples from highly populated residential areas, establishing several drive-through testing stations across the country, mandating and imposing the use of masks and physical distancing in public places, border closures starting on March 19, 2020, and public lockdown and stay at home orders starting on March 23, 2020 were implemented. Relative to the total population, the UAE is one of the top 10 countries in terms of the daily number of tested individuals for COVID-19 [2]. By December 22, 2020, 1,970,552 per one million population COVID-19 RT-PCR tests had been performed in the country [2]. This mass screening has dramatically helped identify more COVID-19 cases. The partial reopening of malls and other public places started on April 23, 2020. The country also adopted gradual reopening of public places and return to work and schools. The implemented strategy for containing the pandemic was successfully able to maintain a low positivity rate, reaching 0.9% by December 21, 2020 [11].

In several studies, the prevalence of asymptomatic COVID-19 positive cases has ranged from 20% to 86%; such asymptomatic cases are defined as individuals with positive viral nucleic acid tests but without any COVID-19 symptoms [8,12–14]. To better understand the identified COVID-19 cases, we characterized COVID-19 cases, described the factors associated with a COVID-19 symptomatic state, and quantified the strength of the association between the symptomatic state and repeated positivity in three subsequent RT-PCR testing rounds during the disease course.

## Materials and methods

This study was approved by the Abu Dhabi Health COVID-19 Research Ethics Committee (IRB DOH/CVDC/2020/1518). Given the retrospective nature of the study, patients’ informed consent was waived. This study followed and was reported according to the Strengthening the Reporting of Observational Studies in Epidemiology (STROBE) reporting guidelines.

### Data source

We reviewed the first cohort of 1,249 RT-PCR-confirmed COVID-19 cases that were passively or actively identified and reported to health authorities in the Emirate of Abu Dhabi, United Arab Emirates, up to April 8, 2020. In the Emirate of Abu Dhabi, the first COVID-19 PCR-confirmed case was reported on February 28, 2020. Information on the reported symptoms was recorded. Self-reported sociodemographic characteristics (age, sex, nationality, and place of work), chronic comorbidities (e.g., DM, hypertension, anemia, and respiratory diseases), and travel history in the past month were also collected. Only RT-PCR-confirmed COVID-19 cases with information on their symptomatic state at the time of the first PCR test during the specified study period were considered eligible and analyzed.

### Specimen collection and viral nucleic acid detection

Upper respiratory tract specimens were collected from both nasopharyngeal or oropharyngeal swabs by trained medical staffs. Viral genome detection was performed using RT-PCR according to the manufacturer’s instructions (BGI Genomics Co. Ltd). According to the local health authorities’ guidelines for repeated testing of positive cases, RT-PCR testing should be repeated every 48–72 h. After the first RT-PCR index test, information on up to three subsequent RT-PCR testing rounds was taken for the COVID-19 cases. RT-PCR re-testing of the COVID-19 cases was performed based on the symptomatic state. The asymptomatic cases were re-tested at least two times after the index test, 1–2 days apart, and then at day 12–13 of isolation whereas symptomatic cases were re-tested more frequently since all of them were admitted to the hospital.

### Evidence synthesis

#### Outcomes of interest

The outcome of interest was the symptomatic state (having symptoms vs not having symptoms) of the COVID-19 confirmed cases. A symptom was defined as the existence of any apparent feature(s) indicating a condition of the disease. The apparent features could be, but were not limited to, fever, cough, headache, shortness of breath, or losing smell or appetite. Information on the symptomatic state was collected at the time of sample collection for the first RT-PCR testing.

#### Exposure variables

We categorized the RT-PCR-confirmed COVID-19 cases based on their symptomatic state into asymptomatic or symptomatic (presented with at least one symptom). The cases were categorized into four age groups (≤20, 21–39, 40–59, or ≥60 years), and to preserve sufficient case numbers in each subcategory, the cases were regrouped into two subgroups based on the nationality (Emirati and non-Emirati). Based on the place of work, they were categorized into four subgroups (not working, working in public places, working in healthcare settings, or working in the aviation and tourism services). This subcategorization was based on the fact that the risk of exposure to COVID-19 and developing symptoms could be associated with the frequency of being exposed to populations with higher risks of transmitting the viral infection such as travelers from highly infected areas. The “not working” category included housewives, children, visitors, and unemployed people. The “working in public places” category included people who worked in shopping markets or malls, delivery services, banks, barbershops, hotels, petrol stations, sales, police and security sectors, and taxi or bus drivers. The healthcare setting category included healthcare staff (physicians, nurses, ambulance drivers, and paramedics), cleaners, and people working in administrative positions (recipients and cashiers). The aviation and tourism services category included people working in airports, travel agencies, and airport taxi and bus drivers.

The self-reported existence of chronic comorbidities (none or at least one comorbidity) and travel history in the past month (yes or no) were each dichotomized into two subcategories. The subcategory ‘at least one comorbidity’ was defined regardless of the type of comorbidity or comorbidities recorded in the same patient.

### Statistical analysis

Continuous variables are presented as the means with standard deviations. Categorical variables are presented as frequencies and proportions. The mean difference in continuous variables between symptomatic and asymptomatic COVID-19 cases was compared using the Student’s *t*-test. Differences in the proportions of the measured categorical variables between symptomatic and asymptomatic cases were assessed with the Chi-square test.

Crude and multivariable binary logistic regression models were used to quantify the strength of the association between the sociodemographic factors and the symptomatic state compared with the asymptomatic state and the repeated positivity of RT-PCR in the subsequent three testing rounds among symptomatic compared with asymptomatic cases. Odds ratios (OR) and adjusted OR (aOR) with 95% confidence interval (CI) were also estimated. To control for any potential confounding effect, all measured exposure variables were included in the multivariable model. Collinearity between exposure variables was investigated using the condition index and the variance inflation factor. No collinearity was present. The maximum variance inflation factor value was 1.3, and the condition index was 9.6.

The proportions of positive conversion (viral shedding) according to symptomatic state were illustrated using the Kaplan–Meier plot, and the log-rank test was used to determine the difference between the two groups. The viral shedding time was calculated from the time of the first RT-PCR test to the negative RT-PCR test result. Statistical analyses were performed using IBM SPSS software (version 26). A p-value of <0.05 was considered statistically significant.

## Results

### Profile of the RT-PCR-confirmed COVID-19 cases

**Fig 1:**
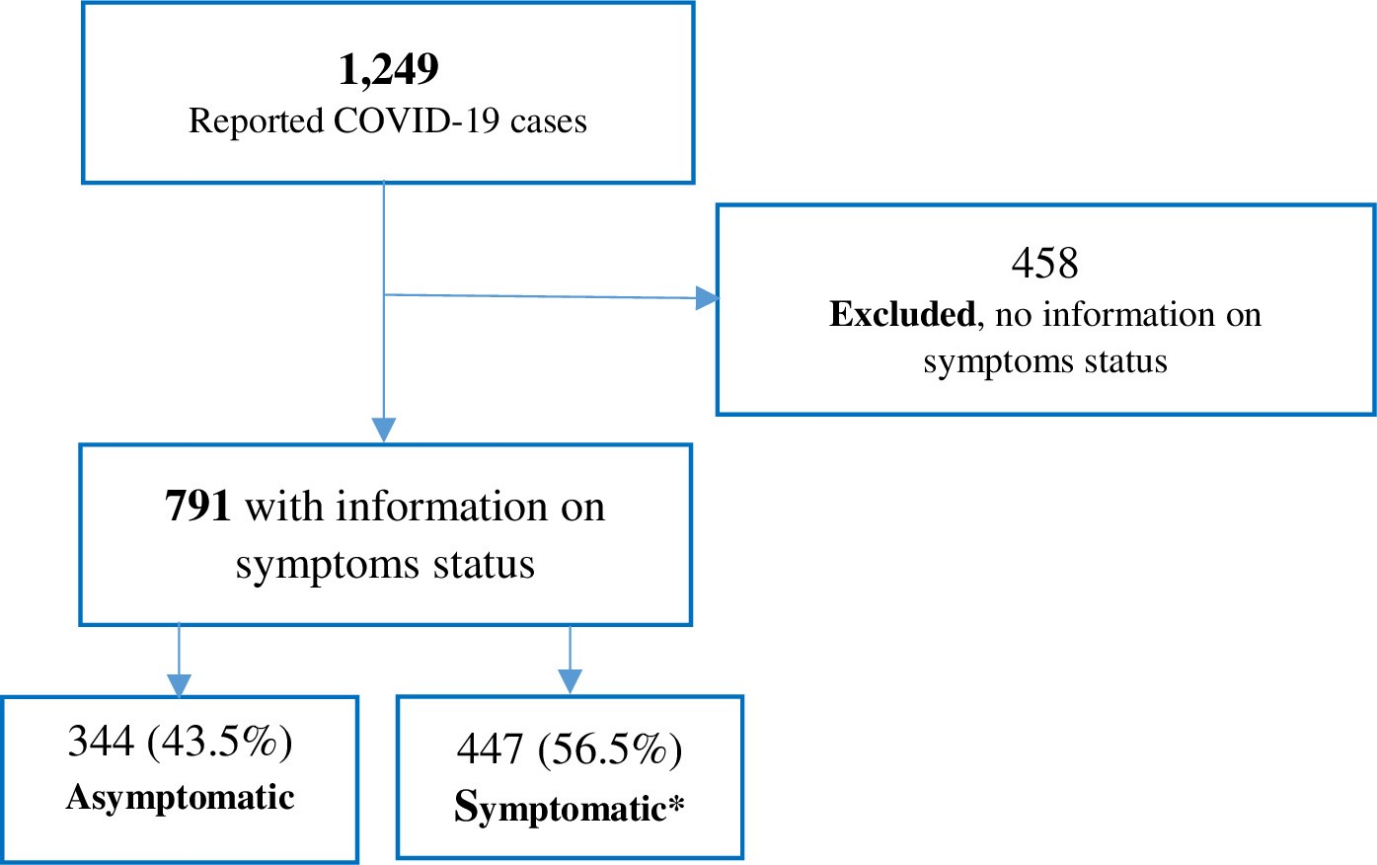
RT-PCR-confirmed COVID-19 cases identified between February 28, 2020 and April 8, 2020 in the Emirate of Abu Dhabi. *mean number of symptoms = 1.75 (range: 1–6 symptoms).

A total of 1,249 RT-PCR-confirmed COVID-19 cases were investigated and reported to the health authorities between February 28 and April 8, 2020. There were missing data on the symptomatic state and other key characteristics in 458 cases. These cases were excluded from the analysis. Among the remaining 791 cases, 43.5% were asymptomatic and 56.5% were symptomatic (Fig 1).

**Fig 2:**
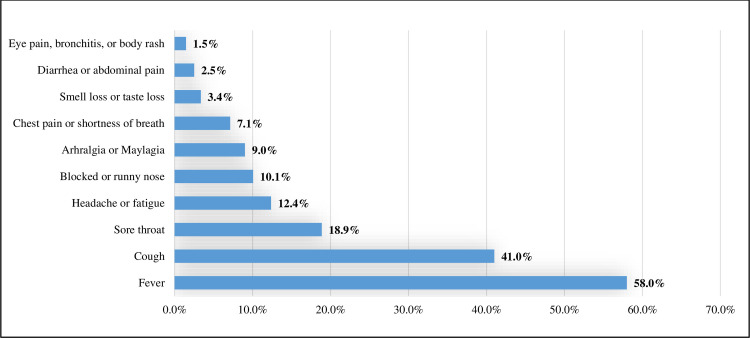
Distribution of symptoms among the 447 symptomatic COVID-19 cases.

Of the 447 symptomatic cases, 47.9% presented with only one symptom, 48.5% presented with 2–3 symptoms, and 3.6% presented with ≥4 symptoms. The most frequent symptom was fever (58.0%), followed by cough (41.0%), sore throat (18.9%), and headache/fatigue (12.4%) (Fig 2).

The mean age of the RT-PCR-confirmed COVID-19 cases was 35.6 ± 12.7 years. Most (79.2%) of the cases were males, 82.2% were Non-Emirati, 61.7% were working in public places, 23.6% had traveled abroad in the past month, and 13.1% reported having at least one chronic comorbidity. There were 46 (5.8%) cases with DM, 45 (5.7%) with hypertension, 14 (1.8%) with asthma, nine (1.1%) with cancer, five (0.6%) with kidney diseases, five (0.6%) with thyroid diseases, one with anemia, and one with allergy.

Of the 791 RT-PCR-confirmed COVID-19 cases, 388 (49.1%) were re-tested (second testing round) a mean of 2.7 ± 1.4 days after the first testing round. Of these 388 cases, 260 (67.0%) were re-tested (third testing round) a mean of 2.4 ± 1.2 days after the second testing round, and of these 260 cases, 62.7% were re-tested (fourth PCR testing) a mean of 3.2 ± 1.8 days after the third testing round (Table 1). Overall, there was a mean of 8.3 ± 2.5 days from the first to the fourth RT-PCR testing rounds. The mean was different between symptomatic (8.7± 2.1 days) and asymptomatic (7.7± 2.6 days) COVID-19 cases (p = 0.040).

**Table 1:** Sociodemographic distribution and subsequent reverse transcriptase polymerase chain reaction (RT-PCR) testing outcomes of the coronavirus disease 2019 (COVID-19) positive confirmed cases by symptomatic state.

Characteristics	All COVID-19 cases n (%)	COVID-19 cases by symptomatic state		
		Asymptomatic (n = 344) n (%)	Symptomatic (n = 447) n (%)	P-value
**Age,** years Mean, 35.6 ± 12.7	791	34.5 ± 12.7	36.3 ± 12.6	0.049
Range = 1–81		1–71	1–81	
≤20	49 (6.3)	28 (8.3)	21 (4.7)	0.040
21–39	479 (61.3)	211 (62.6)	268 (60.2)	
40–59	213 (27.2)	78 (23.1)	135 (30.3)	
≥60	41 (5.2)	20 (5.9)	21 (4.7)	
*Missing*	9	7	2	
**Sex**				0.202
Male	626 (79.2)	279 (81.3)	347 (77.6)	
Female	164 (20.8)	64 (18.7)	100 (22.4)	
*Missing*	1	1		
**Nationality**				0.070
Emirati	141 (17.8)	71 (20.6)	70 (15.7)	
Non-Emirati	650 (82.2)	273 (79.4)	377 (84.3)	
**Place of work**				0.006
Not working^a^	135 (18.0)	72 (22.7)	63 (14.6)	
Public places	488 (65.2)	204 (64.4)	284 (65.7)	
Healthcare setting	58 (7.7)	21 (6.6)	37 (8.6)	
Aviation and tourism services	68 (9.1)	20 (6.3)	48 (11.1)	
*Missing*	42	27	15	
**Chronic comorbidity**				
None	663 (86.4)	303 (90.3)	360 (83.3)	0.004
At least one comorbidity^b^	104 (13.6)	32 (9.6)	72 (16.7)	
*Missing*	24	9	15	
**Travel in the past month**				0.298
No	519 (73.5)	231 (75.5)	288 (72.0)	
Yes	187 (26.5)	75 (24.5)	112 (28.0)	
*Missing*	85	38	47	
**Second PCR test**		N = 159	N = 229	<0.001
Negative	71 (18.3)	43 (27.0)	28 (12.2)	
Positive	317 (81.7)	116 (73.0)	201 (87.8)	
*Not tested*^*c*^	403	185	218	
**Third PCR test**		N = 116	N = 144	0.009
Negative	69 (26.5)	40 (34.5)	29 (20.1)	
Positive	191 (73.5)	76 (65.5)	115 (79.9)	
*Not tested*^*c*^	128	43	85	
**Fourth PCR test**		N = 74	N = 89	0.048
Negative	92 (56.4)	48 (64.9)	44 (49.4)	
Positive	71 (43.6)	26 (35.1)	45 (50.6)	
*Not tested*^*c*^	97	42	55	

%, valid percentage.

^a^ Including children, visitors, students, housewives, and not working people.

^b^ DM (46), hypertension (45), asthma (14), cancer (9), kidney diseases (5), thyroid diseases (5), anemia (1), allergy (1), allowing for overlapping when more than one chronic comorbidity are recorded in the same patient.

^c^ Out of those tested in the previous test.

### Symptomatic versus asymptomatic

The symptomatic cases were significantly older than the asymptomatic cases (mean difference = 1.8 years, p = 0.049). There were more symptomatic cases (35.1%) aged ≥40 years than asymptomatic cases (29.0%). There were also significant differences in the frequency distributions of the place of work between the symptomatic and asymptomatic cases (p = 0.006). More than two-thirds of the symptomatic and asymptomatic (64.4% and 65.7%, respectively) cases were working in public places. Of the RT-PCR-confirmed COVID-19 cases working in healthcare settings and the aviation or tourism services, more were symptomatic (19.7%) than asymptomatic (12.9%). A significantly higher proportion of symptomatic cases had at least one chronic comorbidity than that of asymptomatic cases (16.7% vs 9.6%, respectively). In each of the three subsequent RT-PCR testing rounds, the proportion of those testing positive to COVID-19 was substantially higher among symptomatic cases than among asymptomatic cases (Table 1).

### Crude and independent factors associated with symptomatic state

The RT-PCR-confirmed COVID-19 cases aged 40–59 years had 2.3-fold (95% CI: 1.23–4.34) higher odds of being symptomatic. This association remained insignificant in the multivariable model. Individuals working in public places (aOR: 1.76, 95% CI: 1.11–2.80), in healthcare settings (aOR: 2.09, 95% CI: 1.01–4.31), or in the aviation and tourism services (aOR: 2.24, 95% CI: 1.14–4.40) had a higher likelihood of being symptomatic than not working individuals. Reporting at least one chronic comorbidity was associated with a 1.76-times higher likelihood of having symptoms (aOR: 1.76, 95% CI: 1.03–3.01). Overall, working compared with not working was associated with 80% increased odds of presenting at least one symptom to COVID-19 (aOR: 1.80, 95% CI, 1.16–2.79) (Table 2).

**Table 2:** Factors associated with the symptomatic state of the reverse transcriptase polymerase chain reaction (RT-PCR)-confirmed coronavirus disease 2019 (COVID-19) cases compared with asymptomatic cases.

	OR (95% CI)	aOR (95% CI)
**Age**		
≤20	1.00	1.00
21–39	1.69 (0.94–3.08)	1.41 (0.65–3.08)
40–59	2.31 (1.23–4.34)**	1.48 (0.65–3.38)
≥60	1.40 (0.61–3.22)	0.88 (0.31–2.51)
**Sex**		
Female	1.00	1.00
Male	0.79 (0.56–1.13)	0.93 (0.61–1.40)
**Nationality**		
Emirati	1.00	1.00
Non-Emirati	1.40 (0.97–2.02)	1.44 (0.96–2.17)
**Place of work**		
Not working^a^	1.00	1.00
Public places	1.59 (1.09–2.33)*	1.76 (1.11–2.80)*
Healthcare setting	2.01 (1.07–3.79)*	2.09 (1.01–4.31)*
Aviation and tourism services	2.74 (1.47–5.11)**	2.24 (1.14–4.40)*
Working vs not working^a^	1.61 (1.11–2.34)*	1.80 (1.16–2.79)**
**Chronic comorbidity**		
None	1.00	1.00
At least one comorbidity	1.89 (1.22–2.95)**	1.76 (1.03–3.01)*
**Travel in the past month**		
No	1.00	1.00
Yes	1.20 (0.85–1.68)	1.10 (0.72–1.68)

CI, confidence interval; OR, odds ratio.

aOR: Adjusted for age (as a continuous variable), sex, occupation, chronic conditions, and travel in the past month.

^a^ Including children, visitors, students, housewives, and not working people.

***p < 0.001

**p = 0.001

*p < 0.05.

### Positivity in subsequent RT-PCR testing rounds

**Fig 3:**
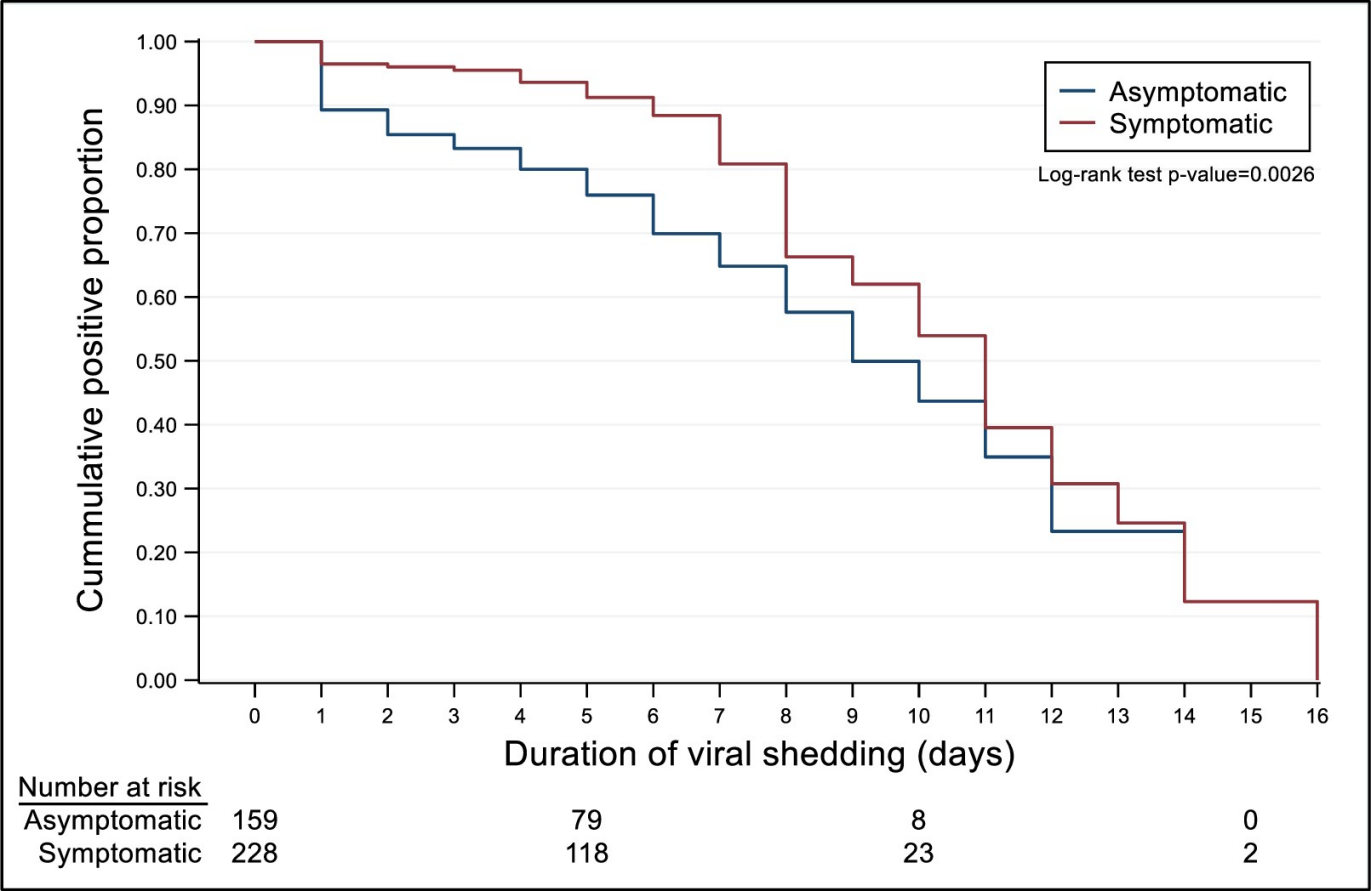
Kaplan–Meier curves for the duration of viral shedding among asymptomatic and symptomatic cases.

Symptomatic state was associated with a prolonged duration of viral shedding compared with asymptomatic state (p for log-rank test = 0.0026) (Fig 3).

In each of the three subsequent RT-PCR testing rounds, symptomatic COVID-19 cases had an over two times greater likelihood of testing positive than asymptomatic cases after adjusting for potential confounders and differences in the mean days of duration between any two subsequent testing rounds (Table 3).

**Table 3:** Crude and adjusted association between symptomatic state and testing positive to coronavirus disease 2019 (COVID-19) in three subsequent reverse transcriptase polymerase chain reaction (RT-PCR) testing rounds.

Symptomatic state	Second PCR test		Third PCR test		Fourth PCR test	
	OR (95% CI)	aOR (95% CI)	OR (95% CI)	aOR (95% CI)	OR (95% CI)	aOR (95% CI)
**Asymptomatic**	1.00	1.00	1.00	1.00	1.00	1.00
**Symptomatic**	2.66 (1.57–4.51)***	2.70 (1.45–5.03)**	2.09 (1.19–3.65)*	2.26 (1.17–4.36)*	1.89 (1.00–3.56)*	2.34 (1.07–5.14)*

CI, confidence interval; OR, odds ratio.

aOR: Adjusted odds ratio for the difference in duration (in days) between the two subsequent PCR tests (as a continuous variable), age (as a continuous variable), sex, nationality, number of symptoms, chronic conditions, place of work, and travel in the past month.

OR and aOR were estimated using a binary logistic regression model.

***p < 0.001

**p = 0.001

*p < 0.05.

## Discussion

The presented findings described RT-PCR-confirmed COVID-19 cases according to their symptomatic state and identified the sociodemographic characteristics associated with presenting symptoms in patients with COVID-19. This study also quantified the strength of the association between testing positive in three subsequent RT-PCR testing rounds after the first test. Older age, working, or having at least one chronic comorbidity was independently associated with developing COVID-19 symptoms. Symptomatic COVID-19 cases were independently more likely to test positive for COVID-19 after an average duration of 8.3 days from the first confirmatory testing round.

The finding that 43.5% of the reported COVID-19 cases were asymptomatic is higher than the previously reported asymptomatic proportions in Columbia (12.0%) [15], on the Diamond Princess cruise in Japan (18.0%) [8], in the Republic of Korea (29.4%) [5], and in Japanese individuals evacuated from Wuhan (31.0%) [7]. The observed higher proportion of asymptomatic COVID-19 cases in the UAE is merely attributed to the expanded active tracing and screening activities implemented by health authorities. As an outbreak containment strategy, a large number of contacts of confirmed COVID-19 cases were traced and tested. The first two reported COVID-19 symptomatic cases on February 28, 2020 were two members of an Italian cycling racing team who had arrived to the UAE on February 20, 2020. From these two cases, 693 individuals were traced and tested for COVID-19 within 1–2 days after identification. Contact tracing and testing can detect a substantial number of potential silent transmitters. The mass tracing and testing activities implemented by the health authorities in the UAE might have contributed to the identification of many COVID-19 carriers who were still in the presymptomatic phase. Relative to the total population, the UAE is one of the top countries with regard to the total number of COVID-19 screening tests performed with over 19.6 million tests were performed in the UAE for COVID-19 as of December 22, 2020 [2].

The crude finding of an inverse association between younger age and presence of COVID-19 symptoms is consistent with a previous report [16]. Younger people are more likely to be healthier and not have underlying comorbidities such as DM and hypertension. However, the disappearance of this significant finding in the multivariate model is a result of the confounding effect of the “working status”. Younger populations are more likely to be engaged in income-generating activities or being students rather than being retired or unemployed. In the present study, the COVID-19 symptomatic state was associated with working in public places (e.g., petrol stations and shopping malls), healthcare settings, or the aviation and tourism sectors (e.g., cabin crews and airport taxi drivers). Repeated exposure to COVID-19 carriers is more likely to occur in working places with greater exposure to the public. Similarly, working in closed spaces including healthcare settings or in aviation and tourism services potentially increases the risk of exposure and re-exposure to virus carriers such as confirmed patients and international travelers. Re-exposure to more COVID-19 carriers or contaminated physical services [17] may have contributed to an increased level of the contracted viral load, which may have expedited the symptomatic state. Nevertheless, this observation does not undermine the role of household transmission among retired or unemployed people [18–20].

COVID-19-positive cases with at least one chronic comorbidity were also positively associated with the symptomatic state. DM and hypertension were the most frequent chronic comorbidities reported in our COVID-19 population. This observation is in line with other reports documenting DM and hypertension as the most distinctive comorbidities in patients with COVID-19 [21–24]. People with chronic comorbidities are more likely to be older and suffer from immune system impairments. Patients with DM suffer from a lack of energy supply to immune cells, which subsequently increases the virulence of infectious microorganisms [25–27]. These impairments weaken the immune system response to microorganisms [28,29]. Hence, individuals with DM are more likely to be infected and are at a higher risk for complications and death from COVID-19 and other pathogens including SARS, MERS [22,30–32], and tuberculosis [27]. A recent study discussed the mechanism by which DM modulates the virus–host interactions and host–immune responses [33]. Following the uptake of COVID-19 by patients with DM, the virus invades the respiratory epithelium and other target cells by binding to cell surface angiotensin-converting enzyme 2, and as a result of the impairment of early recruitment and function of neutrophils and macrophages in patients with DM, the delay in the initiation of adaptive immunity and dysregulation of the cytokine response may lead to the initiation of the cytokine storm, which is associated with the symptomatic state and death among patients with DM. However, in patients with hypertension, antihypertensive drugs such as angiotensin-converting enzyme inhibitors and statins upregulate angiotensin-converting enzyme 2 (ACE2). Increased ACE2 may favor the increased cellular binding of COVID-19 [34–36]. In addition to being associated with progression to the symptomatic state, hypertension and DM delay viral clearance in patients with COVID-19 [37].

In our study, although the symptomatic state was associated with a delay in testing negative in the subsequent testing rounds compared with asymptomatic cases, a substantial proportion of asymptomatic cases also tested positive. In the second RT-PCR testing round, which was performed an average of 2.7 days after the first test, almost three-quarters (73.0%) of the asymptomatic COVID-19 cases tested positive, while over one-third (35.1%) tested positive an average of 8.3 days after the first test. This finding is of paramount public health significance and has implications related to early screening, detection, and timing of quarantine of asymptomatic COVID-19 cases. Asymptomatic and symptomatic patients with COVID-19 were reported to have a similar viral load after a median follow-up of 24 days from diagnosis [5]. The early screening and detection of silent transmitters in the community along with evidence-based quarantine timing would positively contribute to controlling the role of silent transmitters during the current and future pandemic waves.

### Strengths and limitations

There are several potential limitations that should be noted when interpreting our findings. First, there is a risk of reporting bias, which could have underestimated or overestimated our findings. However, this is not likely because all the data were collected by trained healthcare staff who are familiar with data collection and with the definition of chronic comorbidities. Second, our analysis may have been limited because we categorized the COVID-19 cases into symptomatic and asymptomatic states without further categorization into mild, moderate, severe, or critical states. This method was utilized because we lacked the other clinical parameters necessary for such categorization. However, performing further analysis according to the number of symptoms (asymptomatic, one symptom, 2–3 symptoms, and ≥4 symptoms) was consistent with the current findings apart from the significant reduction in the power of the obtained estimates (S1 and S2 Tables). Another important limitation that would potentially further limit the generalizability of our findings is the substantial proportion (36.7%) of missing data on the symptomatic state of the COVID-19 cases. However, the COVID-19 cases included in this analysis were similar to those with missing data, according to the measured exposure variables, except for the number of chronic comorbidities. This difference in the proportion of COVID-19 cases with at least one medical condition could be explained by the observed large proportion of missing data on the self-reported medical conditions of the excluded cases. Furthermore, there was no information on the number of presymptomatic patients who subsequently developed symptoms during observation. Finally, all the included studies relied on RT-PCR testing; no further investigation using chest computed tomography imaging was performed. The effect of this could be bi-directional and result in either overestimation or underestimation because some cases might have been missed due to false-negative results or some cases might have been included due to false-positive COVID-19 results [38,39].

Despite these limitations, to the best of our knowledge, this is the first study to characterize COVID-19-positive cases in the UAE according to the symptomatic state and to shed light on the strength of the association between sociodemographic characteristics and chronic comorbidities with the symptomatic state of COVID-19 cases. Furthermore, our study was based on data collected by trained personnel using a standardized data collection and RT-PCR testing procedure. Our study also provided empirical evidence on the positive association between symptomatic state and delays in testing negative compared with asymptomatic COVID-19 cases.

## Conclusions

A substantial proportion of the COVID-19 cases in the Emirate of Abu Dhabi identified between February 28 and April 8, 2020 were asymptomatic. Working in settings with a higher likelihood of exposure and re-exposure to confirmed or potential virus carriers, or suffering from at least one chronic comorbidity were independently positively associated with the symptomatic state of the COVID-19 cases. The estimated proportion of asymptomatic cases is a vital parameter for future studies; moreover, the estimated strength of the association between identified exposures and the symptomatic state and between testing positive in further RT-PCR testing rounds is vital for public health prevention and control interventions. Further follow-up cohort studies are necessary to provide more insight into viral clearance and clinical outcomes in COVID-19 symptomatic and asymptomatic cases.

## Supporting information

S1 Table Bivariate distribution of coronavirus disease 2019 reverse transcriptase polymerase chain reaction-confirmed cases by symptomatic state. (DOCX)

S2 Table Crude and adjusted strength of the associations between the number of symptoms, number of chronic conditions, and travel history with testing positive to coronavirus disease 2019 in three subsequent polymerase chain reaction testing rounds. (DOCX)
