# Supplementary material for: Epidemiological characterization of symptomatic and asymptomatic COVID-19 cases and positivity in subsequent RT-PCR tests in the United Arab Emirates
Source: PLoS One. 2021 Feb 12;16(2):e0246903. doi: 10.1371/journal.pone.0246903 (PMC7880695; doi:10.1371/journal.pone.0246903)
Supplement: S1 Table — (DOCX) [file pone.0246903.s001.docx]

**S1 Table**

| **Characteristics** | **P-value^1^** | **Asymptomatic**  **%** | **Symptomatic^2^**  **%** | | | | **P-value^3^** |
| --- | --- | --- | --- | --- | --- | --- | --- |
|  |  |  | **All**, % | **One symptom** | **Two symptoms** | **≥3 symptoms** |  |
| **All** |  | 344 | 447 | 214 | 152 | 79 |  |
| **Age**  mean = 35.6 ± 12.7 years  range = 1–81 years | 0.040 | 34.5 ± 12.7 | 36.3 ± 12.6 | 34.8 ± 12.3 | 37.1 ± 13.0 | 38.9 ± 12.2 | 0.027 |
| ≤20 |  | 8.3 | 4.7 | 6.5 | 3.3 | 2.5 | 0.154 |
| 21–39 |  | 62.6 | 60.2 | 60.7 | 62.5 | 54.4 |  |
| 40–59 |  | 23.1 | 30.3 | 29.9 | 27.0 | 38.0 |  |
| ≥60 |  | 5.9 | 4.7 | 2.8 | 7.2 | 5.1 |  |
| *Missing (n = 9)* |  | 7 | 2 |  |  |  |  |
| **Sex** | 0.202 |  |  |  |  |  | 0.005 |
| Male |  | 81.3 | 77.6 | 84.1 | 70.1 | 74.7 |  |
| Female |  | 18.7 | 22.4 | 15.9 | 29.9 | 25.3 |  |
| *Missing (n = 1)* |  |  |  |  |  |  |  |
| **Nationality** | 0.040 |  |  |  |  |  | 0.008 |
| Arab |  | 40.1 | 35.7 | 28.6 | 26.0 | 38.0 |  |
| Asian |  | 51.6 | 50.4 | 62.4 | 55.8 | 43.0 |  |
| Others |  | 8.3 | 13.9 | 8.9 | 18.2 | 19.0 |  |
| *Missing (n = 6)* |  |  |  |  |  |  |  |
| **Place of work** | 0.006 |  |  |  |  |  | 0.102 |
| Public places |  | 64.4 | 65.7 | 72.2 | 61.2 | 56.2 |  |
| Healthcare settings |  | 6.6 | 8.6 | 6.1 | 9.5 | 13.7 |  |
| Aviation and tourism services |  | 6.3 | 11.1 | 8.0 | 14.3 | 13.7 |  |
| Child, visitor, student, not working |  | 22.7 | 14.6 | 13.7 | 15.0 | 16.4 |  |
| *Missing (n* = *42)* |  |  |  |  |  |  |  |
| **Chronic conditions** |  |  |  |  |  |  | 0.735 |
| None | 0.011 | 90.3 | 83.3 | 83.6 | 81.0 | 87.2 |  |
| One condition |  | 7.8 | 12.0 | 12.1 | 12.9 | 10.3 |  |
| ≥2 conditions |  | 1.8 | 4.6 | 4.3 | 6.1 | 2.6 |  |
| At least one condition | 0.004 | 9.6 | 16.7 | 16.4 | 19.0 | 12.8 | 0.487 |
| *Missing (n* = *24)* |  | 9 | 15 |  |  |  |  |
| **Travel in the past month** | 0.298 |  |  |  |  |  | <0.001 |
| No |  | 75.5 | 72.0 | 79.4 | 71.0 | 52.9 |  |
| Yes |  | 24.5 | 28.0 | 20.6 | 29.0 | 47.1 |  |
| *Missing (n* = *85)* |  |  |  |  |  |  |  |
| **Second PCR test** | <0.001 | N = 159 | N = 229 | N = 99 | N = 84 | N = 46 | 0.487 |
| Negative |  | 27.0 | 12.2 | 11.1 | 10.7 | 17.4 |  |
| Positive |  | 73.0 | 87.8 | 88.9 | 89.3 | 82.6 |  |
| *Not tested (n* = *403)* |  |  |  |  |  |  |  |
| **Third PCR test** | 0.009 | N = 116 | N = 144 | N = 60 | N = 52 | N = 32 | 0.438 |
| Negative |  | 34.5 | 20.1 | 18.3 | 17.3 | 28.1 |  |
| Positive |  | 65.5 | 79.9 | 81.7 | 82.7 | 71.9 |  |
| *Not tested (n = 531)* |  |  |  |  |  |  |  |
| **Fourth PCR test** | 0.048 | N = 74 | N = 89 | N = 32 | N = 35 | N = 22 | 0.500 |
| Negative |  | 64.9 | 49.4 | 43.8 | 57.1 | 45.5 |  |
| Positive |  | 35.1 | 50.6 | 56.3 | 42.9 | 54.5 |  |
| *Not tested (n = 628)* |  |  |  |  |  |  |  |

^1^ P-value: assesses differences between symptomatic and asymptomatic

^2^ Mild: only one symptom, moderate: 2–3 symptoms, severe: ≥4 symptoms

^3^ P-value: assesses differences between symptomatic levels.
