# Supplementary material for: Epidemiological characterization of symptomatic and asymptomatic COVID-19 cases and positivity in subsequent RT-PCR tests in the United Arab Emirates
Source: PLoS One. 2021 Feb 12;16(2):e0246903. doi: 10.1371/journal.pone.0246903 (PMC7880695; doi:10.1371/journal.pone.0246903)
Supplement: S2 Table — (DOCX) [file pone.0246903.s002.docx]

**S2 Table**

| **Characteristics** | **Second PCR test** | | | **Third PCR test** | | | **Fourth PCR test** | | |
| --- | --- | --- | --- | --- | --- | --- | --- | --- | --- |
|  | **OR (95% CI)** | **aOR (95% CI)^1^** | **aOR (95% CI)^2^** | **OR (95% CI)** | **aOR (95% CI)^1^** | **aOR (95% CI)^2^** | **OR (95% CI)** | **aOR (95% CI)^1^** | **aOR (95% CI)^2^** |
| **Number of symptoms** |  |  |  |  |  |  |  |  |  |
| Asymptomatic | 1.00 | 1.00 | 1.00 | 1.00 | 1.00 | 1.00 | 1.00 | 1.00 | 1.00 |
| One symptom | 2.97 (1.45–6.08)^**^ | 3.35 (1.51–7.46)^*^ | 3.42 (1.51-7.75)^*^ | 2.34 (1.10–5.00)^*^ | 2.59 (1.10–6.08)^*^ | 2.92 (1.1-7.07)^*^ | 2.37 (1.02–5.53)^*^ | 2.81 (1.06–7.42)^*^ | 2.88 (1.09–7.70)^*^ |
| Two symptoms | 3.09 (1.42–6.71)^**^ | 2.84 (1.19–6.76)^*^ | 2.55 (1.03-6.33)^*^ | 2.52 (1.11–5.67)^*^ | 3.17 (1.23–8.13)^*^ | 3.06 (1.19-7.87)^*^ | 1.39 (0.61–3.15) | 1.65 (0.61–4.46) | 1.74 (0.63-4.85) |
| ≥3 symptoms | 1.76 (0.76–4.07) | 1.75 (0.69–4.43) | 1.93 (0.75-5.00) | 1.35 (1.11–5.67) | 1.62 (0.64–4.12) | 1.54 (0.60-3.93) | 2.22 (0.84–5.82) | 2.30 (0.77–6.93) | 2.40 (0.79–7.29) |
| At least one symptom | 2.66 (1.57–4.51)^***^ | 2.72 (1.49–4.95)^**^ | 2.70 (1.45-5.03)^**^ | 2.09 (1.19–3.65)^*^ | 2.40 (1.26–4.60)^**^ | 2.26 (1.17-4.36)^*^ | 1.89 (1.00–3.56)^*^ | 2.23 (1.04–4.80)^*^ | 2.34 (1.07–5.14)^*^ |
| At least one symptom, excluding 3 pregnant women | 2.66 (1.57–4.51)^***^ | 2.72 (1.49–4.95)^**^ | 2.71 (1.50–4.92)^**^ | 2.09 (1.19–3.65)^*^ | 2.40 (1.26–4.60)^**^ | 2.51 (1.32–4.77)^**^ | 1.89 (1.00–3.56)^*^ | 2.23 (1.04–4.80)^*^ | 2.34 (1.07–5.14)^*^ |

OR: odds ratio; PCR: polymerase chain reaction

^1^ aOR, adjusted odds ratio for age (as a continuous variable), sex, nationality, number of symptoms, chronic conditions, place of work, and travel history.

^2^ OR, adjusted odds ratio for the difference in duration (in days) between the two subsequent PCR tests (as a continuous variable), age (as a continuous variable), sex, nationality, number of symptoms, chronic conditions, place of work, and travel history.

^***^p < 0.001, ^**^p = 0.001, ^*^p < 0.05

**Mean duration in days:**

First and second PCR tests (n = 388): 2.7 ± 1.4 days

Second and third PCR tests (n = 258): 2.4 ± 1.2 days

Third and fourth PCR tests (n = 163): 3.2 ± 1.8 days

**B**

**A**
